# Investigation of neural stem cell-specific regulatory promoter elements

**DOI:** 10.3892/etm.2012.614

**Published:** 2012-06-18

**Authors:** HONGHUA YUAN, ANKANG HU, LI ZHANG, XIAORONG ZHU

**Affiliations:** 1 Department of Neurobiology and; 2 Laboratory Animal Center, Xuzhou Medical College, Xuzhou, Jiangsu 221002, P.R. China

**Keywords:** nestin gene, promoter, regulation

## Abstract

The present study aimed to investigate neural stem cell (NSC)-specific regulatory promoter elements. PCR was employed to amplify the full sequence (4,000 bp) and core sequence (400 bp) of the promoter and intron-2 of the mouse nestin gene. pcDNA3.1 was used as a template to construct 6 different recombinant plasmids. CMV, CMV + intron-2, the full sequence of the nestin gene promoter, the full sequence of the nestin gene promoter + intron-2, the core sequence of the nestin gene promoter and the core sequence of the nestin gene promoter + intron-2 were independently used as promoters to regulate EGFP expression. The 6 recombinant plasmids were independently used to transfect nestin-positive and nestin-negative cells, and the expression of EGFP was observed under a fluorescence microscope. At the same time, flow cytometry was carried out to measure the proportion of cells positive for EGFP. The results showed that the full sequence and core sequence of the nestin gene promoter non-specifically regulated EGFP expression in cells and exhibit potent regulatory potency. The full sequence or core sequence of the nestin gene promoter which was fused with intron-2 can only regulate the EGFP expression in nestin-positive cells. CMV + intron-2 have non-specific regulation of EGFP alone. Thus, we conclude that the full sequence of the nestin gene promoter which is fused with intron-2 can specifically regulate the expression of exogenous genes in nestin-positive cells.

## Introduction

In 1992, Reynolds and Weiss (1) for the first time separated neural stem cells (NSCs) from the subventricular zone (SVZ) of mice. Since then, NSCs with the potential for self-renew and differentiation into neurons, astrocytes and oligodendrocytes have been identified in the subgranular zone (SGZ) of adult rats, non-human primates and humans. These residual NSCs live in a special microenvironment and are crucial for the maintenance of neurogenesis in the brain of adults (2). The physiological functions of adult NSCs are affected by genes, growth factors, neurotransmitters, stress, steroids, age and environment. Regulation of intracranial adult NSCs may promote the proliferation and differentiation of NSCs and the migration of NSCs into target sites, which may be beneficial for the repair of damaged neurons and the subsequent improvement of neurological diseases. At present, studies are increasing their focus on the role of genes in the regulation of NSCs. To obtain a promoter that can regulate the expression of exogenous genes in NSCs is critical for gene regulation. Nestin is specifically expressed in NSCs (3). However, the promoter has the potential of only non-specific regulation (4). The intron-2 of the nestin gene is a promoter specific to neural precursor cells in the nervous system (5). In the present study, the promoter sequence of the nestin gene was fused with intron-2 and the regulatory potential of the fusion gene in NSCs was investigated.

## Materials and methods

### Main reagents and instruments

Saturated chloroform, proteinase K, primers, restriction enzymes (*Hin*dIII, *Not*I, *Bam*HI, *Mlu*I), T4 DNA ligase reaction mixture (Shanghai Sangon Biological Engineering Technology and Services Co, Ltd., Shanghai, China), PrimeSTAR^™^HS DNA polymerase (Takara Bio, Inc.), EndoFree Plasmid Maxi kit, QIAquick gel extraction kit (Qiagen), SV Minipreps DNA purification system (Promega), Lipofectamine^™^ 2000 reagent (Invitrogen), DMEM, RPMI-1640 (Gibco-BRL), calf serum (Hangzhou Sijiqing Biological Engineering Materials Co., Ltd.), COS-7 cells, C6 cells (Cell Bank of Chinese Academy of Sciences, Shanghai Institute of Cell Biology, Shanghai, China), MEF cells, plasmids (pEGFP-N1 and pcDNA3) and DH5a were used in the present study. TC2323 CO_2_ incubator (Sheldon Manufacturing, Inc., USA), IX70-S8F23 inverted phase contrast microscope (Olympus, Japan), PTC-100 PCR instrument (MJ Research, Inc., USA), BH2-RFL-3T fluorescence microscope (Olympus, Japan) and BD FACalibur flow cytometer (BD Biosciences, USA) were also used in the present study.

### Primer design

The primers were designed according to the promoter sequence of the nestin gene in GenBank. NF1 and NR were primers for the full sequence of the nestin promoter (4,000 bp), and NF2 and NR for the core sequence of the nestin promoter (390 bp): NF1, 5′-CATACGCGTGAGCTCCCT AAACCTATCCCC-3′ (*Mlu*I); NR, 5′-AGCAAGCTTAA GCGGACGTGGAGCACTAG-3′ (*Hin*dIII); NF2, 5′-CATACG CGTTCCCTGAGACCTGCCTGATC-3′ (*Mlu*I). The primers for intron-2 of the nestin gene were designed according to the sequence of nestin in the Genbank. The anticipated length of intron-2 following amplification was 1680 bp: HF, CTAGGA TCCGTACACAGTACTGACTGTCTCCTTG (*Bam*Hl); HR, CATACGCGTGTTGCATGTCCTGCCACTGCAGGATC (*Mlu*I).

### Amplification, retrieval and sequencing of the target genes

DNA was extracted as template DNA from the tails of specific pathogen-free C57BL/6J mice (SCXK[Jiangzu]2010-0003) by using the phenol-chloroform extraction method. The PrimeSTAR HS DNA polymerase was used in the PCR assay for the amplification of the full sequence and core sequence of the nestin promoter and intron-2 of the nestin gene. The PCR products were subjected to 1% agarose (containing 0.1 *μ*g/ml ethidium bromide) electrophoresis, and the bands were visualized under a gel analysis system. Qiagen gel retrieval kit was used for the retrieval of products. The products were sequenced by Shanghai Sangong Pharmaceutical Co., Ltd.

### Construction of the recombinant expression vector

**Figure 1:**
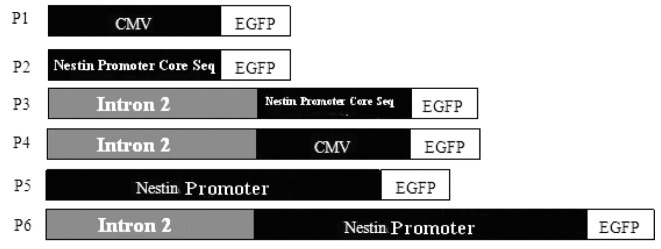
Diagram of recombinant plasmids.

pcDNA3.1 was used as a template for the construction of recombinant plasmids which are shown in Fig. 1.

*P1 plasmid.* The EGFP fragment was collected from the pEGFP-N1 plasmid after digestion with *Hin*dIII and *Not*I. At the same time, the pcDNA3.1 plasmid was digested with *Hin*dIII and *Not*I. The EGFP was ligated to the fragments from pcDNA3.1 by using T4 DNA ligase forming pcDNA3-EGFP which was used for genetic transformation. Plasmid extraction was performed with the Minipreps DNA purification system and the products were identified by *Hin*dIII and *Not*I.

*P2 plasmid.* The P1 plasmid was digested with *Hin*dIII and *Mlu*I and the band at 5,500 bp was retrieved. At the same time, the core sequence of the nestin promoter was collected following digestion with *Hin*dIII and *Mlu*I. The collected fragments were ligated followed by transformation. Plasmid extraction was performed with the Minipreps DNA purification system and the products were identified by *Hin*dIII and *Mlu*I.

*P3 plasmid.* The P1 plasmid was digested with *Bgl*II and *Mlu*I and the band at 5,700 bp was retrieved. At the same time, the intron-2 of the nestin gene was collected following digestion with *Bam*Hl and *Mlu*I. The collected fragments were ligated followed by transformation. Plasmid extraction was performed with the Minipreps DNA purification system and PCR was performed for the amplification of intron-2. The products were identified by *Mlu*I.

*P4 plasmid.* The P2 plasmid was digested with *Bgl*II and *Mlu*I and the band at 6,000 bp was retrieved. At the same time, the intron-2 of the nestin gene was collected following digestion with *Bam*Hl and *Mlu*I. The collected fragments were ligated followed by transformation. Plasmid extraction was performed with the Minipreps DNA purification system and PCR was performed for the amplification of intron-2. The products were identified by *Mlu*I.

*P5 plasmid.* The nestin gene was digested with *Hin*dIII and *Mlu*I and then collected. The P1 plasmid was digested with two restriction enzymes and the band at 5,800 bp was retrieved. The collected fragments were ligated followed by transformation. Plasmid extraction was performed with the Minipreps DNA purification system and the products were identified by *Hin*dIII and *Mlu*I.

*P6 plasmid.* The nestin gene was digested with *Hin*dIII and *Mlu*I and then collected. The P4 plasmid was digested with two restriction enzymes and the band at 5,800 bp was retrieved. The collected fragments were ligated followed by transformation. Plasmid extraction was performed with the Minipreps DNA purification system and the products were identified by *Hin*dIII and *Mlu*I.

### Identification of the transfected cells by fluorescence staining

C6, COS-7 and MEF cells were used for transfection. In brief, the cells were washed in 0.01 mol/l PBS (pH 7.4) thrice (5 min for each) and then fixed in 4% paraformaldehyde at 4°C for 20 min. Then, the solution was removed and the cells were dried for 0.5 h in air. After washing in 0.01 mol/l PBS (pH 7.4) thrice (5 min for each), cells were blocked in 5% normal goat serum containing 0.1% Triton X-100 for 1 h at room temperature and then with an antibody against nestin (1:1,000) at 4°C overnight. After being kept at room temperature for 0.5 h, cells were washed in 0.01 mol/l PBS (pH 7.4) thrice (5 min for each) and then treated with fluorescence-conjugated secondary antibody (1:200) at 4°C overnight. After being kept at room temperature for 0.5 h, cells were washed in 0.01 mol/l PBS (pH 7.4) thrice (5 min for each) and observed under a fluorescence microscope.

### Expression of the target gene in cells transfected with recombinant plasmid

Six different recombinant plasmids were prepared with the EndoFree Plasmid Maxi kit and then used to transfect MEF, C6 and COS-7 cells, independently, by using Lipofectamine^™^ 2000 reagent. Transfection was performed in triplicate (6-well plate) 4 times. Forty-eight hours after transfection, representative images were captured under a fluorescence microscope. Then, cells were washed in PBS twice (3 min for each) and then digested in trypsin followed by cell collection through centrifugation at 1,200 rpm for 5 min. The cells in each well were diluted with 400 *μ*l of PBS and subjected to flow cytometry for the analysis of EGFP-positive cells.

### Statistical analysis

Statistical analysis was carried out with SPSS. Data were expressed as mean ± SD. One-way analysis of variance (ANOVA) was employed to analyze the intra-group difference and least significant difference test to analyze the inter-group difference. A value of P<0.05 was considered statistically significant.

## Results

### PCR amplification

**Figure 2:**
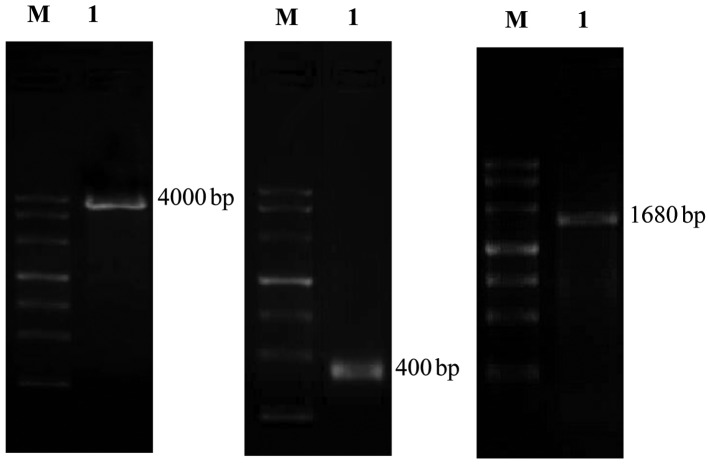
PCR amplification. Left, full sequence of the nestin promoter; middle, core sequence of the nestin promoter; right, intron-2 of the nestin gene. Lane M, maker; lane 1, product of the PCR assay.

Following amplification of the core and full sequence of the nestin promoter by PCR, the products were identified at 4,000 and 400 bp and then retrieved for sequencing (Fig. 2). Results showed that the size and base composition of these products were identical to those published in GenBank. The products of PCR assay were identified at 1,680 bp and then retrieved for sequencing (Fig. 2). Results showed that the size and base composition of these products were identical to those published in GenBank.

### Expression of EGFP in the COS-7 cells transfected with the different recombinant plasmids

**Figure 3:**
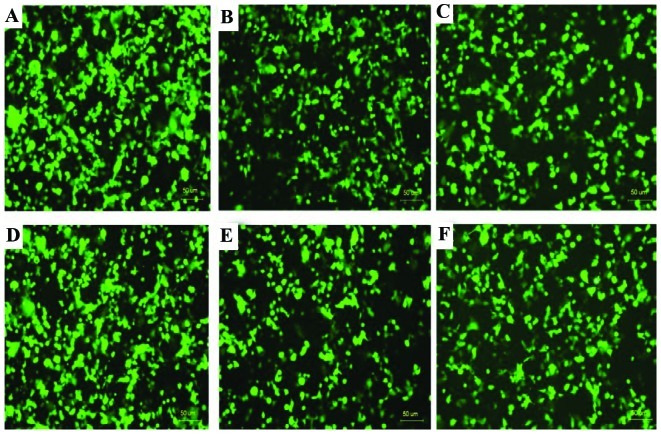
EGFP expression in COS-7 cells transfected with the different recombinant pasmids. (A) COS-7 cells transfected with the P1 plasmid, (B) the P2 plasmid, (C) the P3 plasmid, (D) the P4 plasmid, (E) the P5 plasmid, and (F) the P6 plasmid.

Six different recombinant plasmids were used to transfect COS-7 cells independently. The promoters in the six recombinant plasmids regulated the EGFP expression in COS-7 cells (Fig. 3). Flow cytometry showed that the expression rate of EGFP in COS-7 cells transfected with P1, P2, P3, P4, P5 and P6 plasmids to be 86.3±1.5, 82.1±2.4, 78.0±3.2, 85.3±4.1, 79.6±4.5 and 78.3±4.6%, respectively, showing no significant difference.

### Expression of EGFP in the C6 cells transfected with the different recombinant plasmids

Six different recombinant plasmids were used to transfect C6 cells independently. The promoters in the six recombinant plasmids regulated the EGFP expression in COS-7 cells. Flow cytometry showed that the expression rate of EGFP in COS-7 cells transfected with P1, P2, P3, P4, P5 and P6 plasmids to be 37.5±1.1, 23.1±1.6, 17.1±1.5, 26.0±1.4, 18.3±1.5 and 17.8±1.3%, respectively. The EGFP expression in the P3 group was significantly lower than that in the P1 and P4 groups, but there was no marked difference among the P2, P3, P5 and P6 groups.

### Expression of EGFP in the MEF cells transfected with different recombinant plasmids

Six different recombinant plasmids were used to transfect MEF cells independently. The promoters in six recombinant plasmids regulated EGFP expression in the MEF cells. Flow cytometry showed the expression rate of EGFP in the MEF cells transfected with P1, P2, P3, P4, P5 and P6 plasmids to be 4.4±0.3, 3.9±0.4, 0.1±0.5, 3.9±0.4, 3.7±0.6 and 0.2±0.4%, respectively. The EGFP expression in the P3 and P6 groups was significantly lower than that in the remaining groups.

## Discussion

Studies on the regulation of gene expression may provide clues for the investigation of gene modification. The regulation of gene expression can occur at different levels including replication, amplification, gene activation, transcription, post-transcription, translation and post-translation. The initiation of transcription is a basic control point in the regulation of gene expression. Thus, the promoter of the target gene is critical for the regulation of gene expression and particularly the regulation of transcription (6).

Eukaryotic genes include a core element and an upstream promoter element (7). In the core sequence, the TATA box mediates the formation of transcription-initiation complexes involving RNA polymerase II (Pol II) and the initiation of basic transcription. It also mediates the regulation of downstream elements and determines the site where transcription starts. Based on the analysis using TFSEARCH software, the promoter of the cloned nestin gene has the TATA-like box. The functions of promoters of eukaryotic genes depend on not only the TATA box, but also on one or more upstream regulation elements (UPE). The core sequence of the nestin promoter has the binding sites of basic regulation elements including GATA-1, GATA-3, AP1, AP3 and SP1, which assures that the core sequence of the nestin promoter has the characteristics of the promoter of eukaryotic genes (8).

Results showed that the promoter of the nestin gene had the regulation potential in nestin-positive and nestin-negative cells. To investigate the possibility of cell-specific regulation elements in the 5′ upstream of the mouse nestin gene, we prepared the full sequence (4,000 bp) and core sequence of the nestin promoter (400 bp) as promoters to regulate GEFP expression in different cells. Results showed that the two promoters had non-specific regulations and the activities of the promoters were comparable with that of the CMV promoter. Therefore, we speculated that there were no cell-specific regulation elements in the promoter of the nestin gene. Our results also demonstrated that the full sequence and core sequence of the mouse nestin promoter had the activity of transcription initiation in different mammalian cell lines and could regulate the expression of reporter genes or exogenous genes in different cell lines.

The intron-2 of the mouse nestin gene was 1,686 bp in length and has 51.4 and 81.1% homology to the intron-2 of human and rat nestin genes, respectively (http://seqtool.sdsc.edu). Josephson *et al* (9) confirmed that the POU site in the intron-2 of the rat nestin gene was essential for the normal expression of the nestin gene in the whole central nervous system (CNS), and that the HRE/RXR/TTF1 sites were only found in a few regions of the CNS including the forebrain and dorsal mesencephalon. Tanaka *et al* (10) found that Sox could coordinate with transcription factors in the POU family to regulate nestin expression during neurogenesis. This coordination occurred between class III, POU factors in Brn2 and SoxB1 subfamily (Sox1, Sox2 and Sox3) and Sox11, as well as between Sox2, class III POU factors (Brn1, Brn2, Brn4 and Oct6) and class V POU factor Oct4. The site 1,387–1,396 bp of intron-2 of the mouse nestin gene is a binding site of transcription factors in the Sox family, the site of 1,402–1,410 bp is a binding site of POU factors and the site of 1,435–1,443 bp is a binding site of HRE/RXR/TTF1. Results showed that the intron-2 of the mouse nestin which was fused with the promoter of the nestin gene specifically regulated EGFP expression in nestin-positive cells, while intron-2 which was fused with the promoter CMV had no specific regulation. These findings suggest that, in NSCs, the promoter of the nestin gene can coordinate with intron-2 of the nestin gene to specifically regulate protein expression in NSCs.

Our findings confirm that the promoter of the nestin gene can coordinate with intron-2 of the nestin gene to regulate the expression of exogenous genes in nestin-positive cells. In the CNS, nestin is a NSC-specific protein. The promoter can coordinate with intron-2 to regulate the expression of exogenous genes in NSCs. Our findings may provide evidence for studies on the gene regulation as well as the targeting and positioning of drugs in NSCs.
